# Supplementary figures and images for: 3D-MRI brain glioma intelligent segmentation based on improved 3D U-net network
Source: PLoS One. 2025 Jun 13;20(6):e0325534. doi: 10.1371/journal.pone.0325534 (PMC12165427; doi:10.1371/journal.pone.0325534)

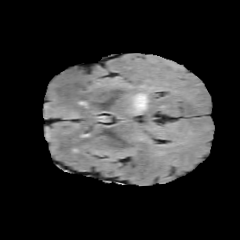

Supplement: S1 Data — (ZIP) [file pone.0325534.s001.zip › Partial data -Supporting Information file/Brats_1/Brats_flair_slice_77.png]

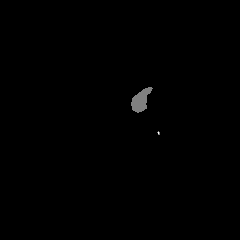

Supplement: S1 Data — (ZIP) [file pone.0325534.s001.zip › Partial data -Supporting Information file/Brats_1/Brats_seg_slice_77.png]

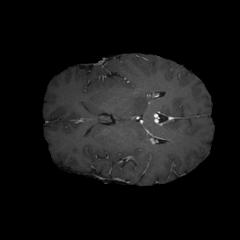

Supplement: S1 Data — (ZIP) [file pone.0325534.s001.zip › Partial data -Supporting Information file/Brats_1/Brats_t1ce_slice_77.png]

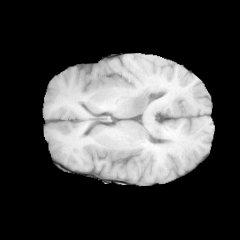

Supplement: S1 Data — (ZIP) [file pone.0325534.s001.zip › Partial data -Supporting Information file/Brats_1/Brats_t1_slice_77.png]

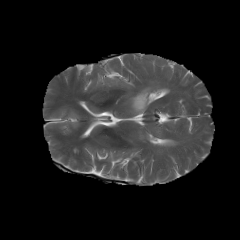

Supplement: S1 Data — (ZIP) [file pone.0325534.s001.zip › Partial data -Supporting Information file/Brats_1/Brats_t2_slice_77.png]

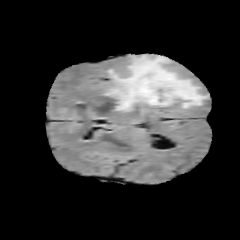

Supplement: S1 Data — (ZIP) [file pone.0325534.s001.zip › Partial data -Supporting Information file/Brats_10/Brats_flair_slice_77.png]

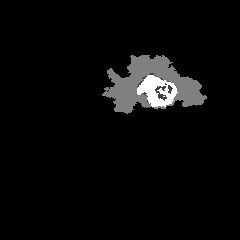

Supplement: S1 Data — (ZIP) [file pone.0325534.s001.zip › Partial data -Supporting Information file/Brats_10/Brats_seg_slice_77.png]

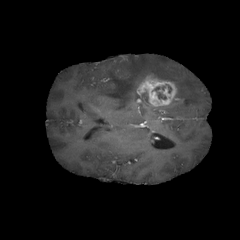

Supplement: S1 Data — (ZIP) [file pone.0325534.s001.zip › Partial data -Supporting Information file/Brats_10/Brats_t1ce_slice_77.png]

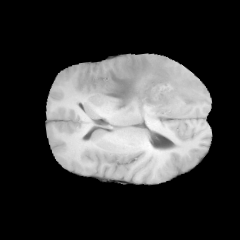

Supplement: S1 Data — (ZIP) [file pone.0325534.s001.zip › Partial data -Supporting Information file/Brats_10/Brats_t1_slice_77.png]

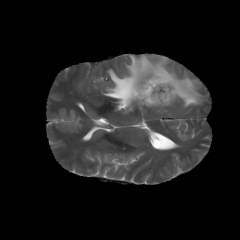

Supplement: S1 Data — (ZIP) [file pone.0325534.s001.zip › Partial data -Supporting Information file/Brats_10/Brats_t2_slice_77.png]

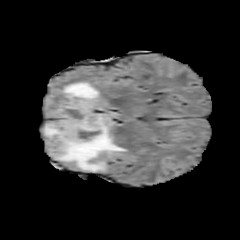

Supplement: S1 Data — (ZIP) [file pone.0325534.s001.zip › Partial data -Supporting Information file/Brats_11/Brats_flair_slice_77.png]

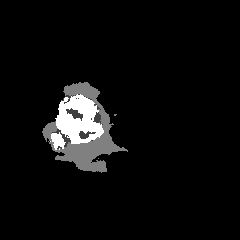

Supplement: S1 Data — (ZIP) [file pone.0325534.s001.zip › Partial data -Supporting Information file/Brats_11/Brats_seg_slice_77.png]

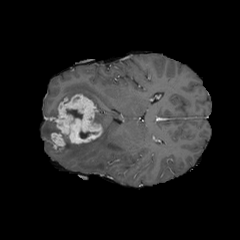

Supplement: S1 Data — (ZIP) [file pone.0325534.s001.zip › Partial data -Supporting Information file/Brats_11/Brats_t1ce_slice_77.png]

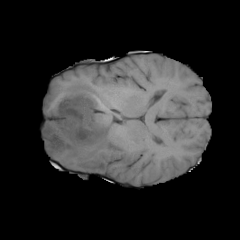

Supplement: S1 Data — (ZIP) [file pone.0325534.s001.zip › Partial data -Supporting Information file/Brats_11/Brats_t1_slice_77.png]

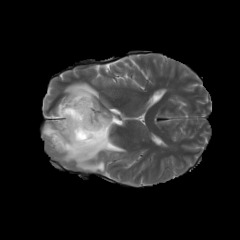

Supplement: S1 Data — (ZIP) [file pone.0325534.s001.zip › Partial data -Supporting Information file/Brats_11/Brats_t2_slice_77.png]

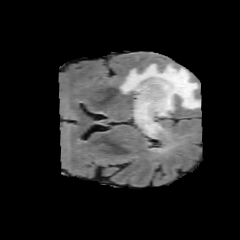

Supplement: S1 Data — (ZIP) [file pone.0325534.s001.zip › Partial data -Supporting Information file/Brats_12/Brats_flair_slice_77.png]

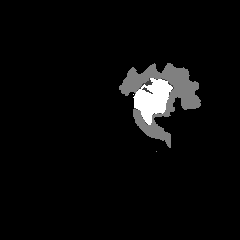

Supplement: S1 Data — (ZIP) [file pone.0325534.s001.zip › Partial data -Supporting Information file/Brats_12/Brats_seg_slice_77.png]

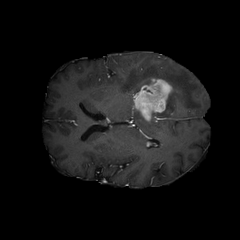

Supplement: S1 Data — (ZIP) [file pone.0325534.s001.zip › Partial data -Supporting Information file/Brats_12/Brats_t1ce_slice_77.png]

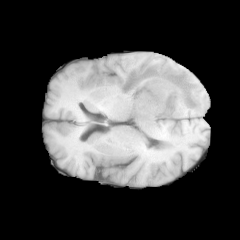

Supplement: S1 Data — (ZIP) [file pone.0325534.s001.zip › Partial data -Supporting Information file/Brats_12/Brats_t1_slice_77.png]

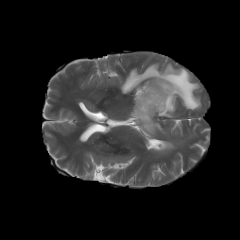

Supplement: S1 Data — (ZIP) [file pone.0325534.s001.zip › Partial data -Supporting Information file/Brats_12/Brats_t2_slice_77.png]

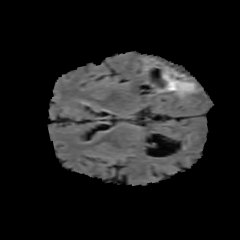

Supplement: S1 Data — (ZIP) [file pone.0325534.s001.zip › Partial data -Supporting Information file/Brats_13/Brats_flair_slice_77.png]

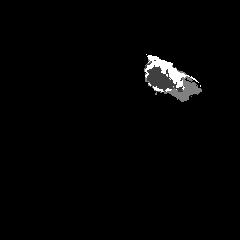

Supplement: S1 Data — (ZIP) [file pone.0325534.s001.zip › Partial data -Supporting Information file/Brats_13/Brats_seg_slice_77.png]

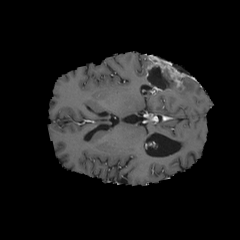

Supplement: S1 Data — (ZIP) [file pone.0325534.s001.zip › Partial data -Supporting Information file/Brats_13/Brats_t1ce_slice_77.png]

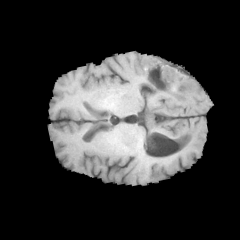

Supplement: S1 Data — (ZIP) [file pone.0325534.s001.zip › Partial data -Supporting Information file/Brats_13/Brats_t1_slice_77.png]

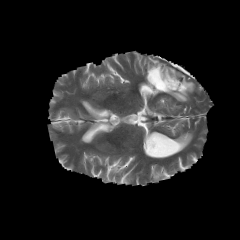

Supplement: S1 Data — (ZIP) [file pone.0325534.s001.zip › Partial data -Supporting Information file/Brats_13/Brats_t2_slice_77.png]

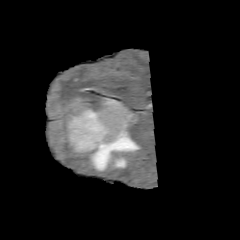

Supplement: S1 Data — (ZIP) [file pone.0325534.s001.zip › Partial data -Supporting Information file/Brats_14/Brats_flair_slice_77.png]

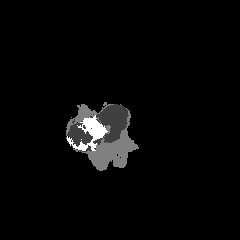

Supplement: S1 Data — (ZIP) [file pone.0325534.s001.zip › Partial data -Supporting Information file/Brats_14/Brats_seg_slice_77.png]

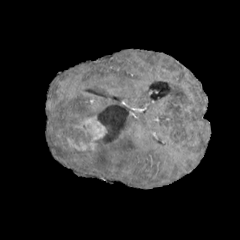

Supplement: S1 Data — (ZIP) [file pone.0325534.s001.zip › Partial data -Supporting Information file/Brats_14/Brats_t1ce_slice_77.png]

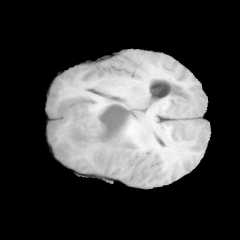

Supplement: S1 Data — (ZIP) [file pone.0325534.s001.zip › Partial data -Supporting Information file/Brats_14/Brats_t1_slice_77.png]

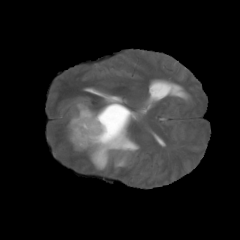

Supplement: S1 Data — (ZIP) [file pone.0325534.s001.zip › Partial data -Supporting Information file/Brats_14/Brats_t2_slice_77.png]

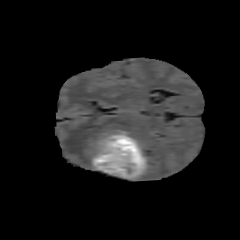

Supplement: S1 Data — (ZIP) [file pone.0325534.s001.zip › Partial data -Supporting Information file/Brats_15/Brats_flair_slice_77.png]

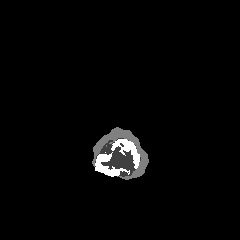

Supplement: S1 Data — (ZIP) [file pone.0325534.s001.zip › Partial data -Supporting Information file/Brats_15/Brats_seg_slice_77.png]

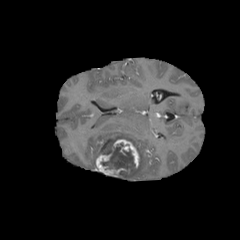

Supplement: S1 Data — (ZIP) [file pone.0325534.s001.zip › Partial data -Supporting Information file/Brats_15/Brats_t1ce_slice_77.png]

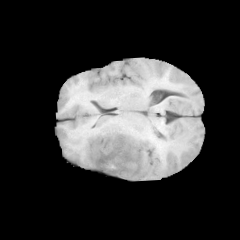

Supplement: S1 Data — (ZIP) [file pone.0325534.s001.zip › Partial data -Supporting Information file/Brats_15/Brats_t1_slice_77.png]

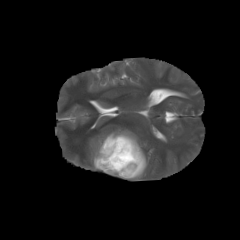

Supplement: S1 Data — (ZIP) [file pone.0325534.s001.zip › Partial data -Supporting Information file/Brats_15/Brats_t2_slice_77.png]

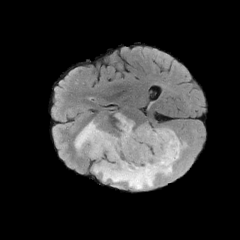

Supplement: S1 Data — (ZIP) [file pone.0325534.s001.zip › Partial data -Supporting Information file/Brats_16/Brats_flair_slice_77.png]

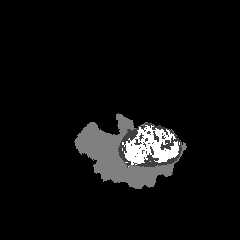

Supplement: S1 Data — (ZIP) [file pone.0325534.s001.zip › Partial data -Supporting Information file/Brats_16/Brats_seg_slice_77.png]

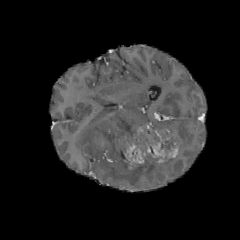

Supplement: S1 Data — (ZIP) [file pone.0325534.s001.zip › Partial data -Supporting Information file/Brats_16/Brats_t1ce_slice_77.png]

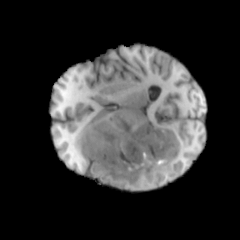

Supplement: S1 Data — (ZIP) [file pone.0325534.s001.zip › Partial data -Supporting Information file/Brats_16/Brats_t1_slice_77.png]

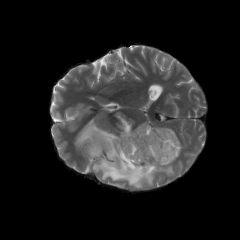

Supplement: S1 Data — (ZIP) [file pone.0325534.s001.zip › Partial data -Supporting Information file/Brats_16/Brats_t2_slice_77.png]

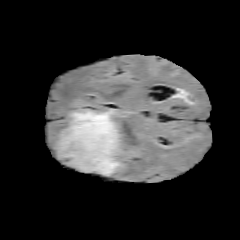

Supplement: S1 Data — (ZIP) [file pone.0325534.s001.zip › Partial data -Supporting Information file/Brats_17/Brats_flair_slice_77.png]

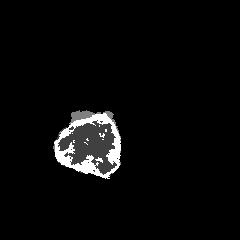

Supplement: S1 Data — (ZIP) [file pone.0325534.s001.zip › Partial data -Supporting Information file/Brats_17/Brats_seg_slice_77.png]

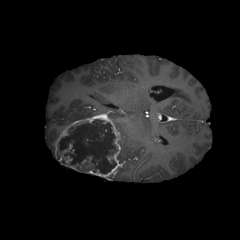

Supplement: S1 Data — (ZIP) [file pone.0325534.s001.zip › Partial data -Supporting Information file/Brats_17/Brats_t1ce_slice_77.png]

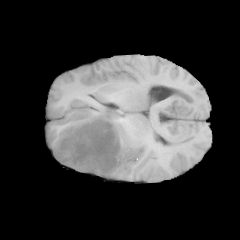

Supplement: S1 Data — (ZIP) [file pone.0325534.s001.zip › Partial data -Supporting Information file/Brats_17/Brats_t1_slice_77.png]

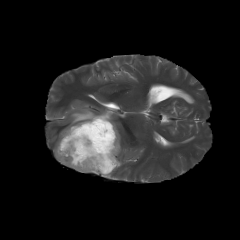

Supplement: S1 Data — (ZIP) [file pone.0325534.s001.zip › Partial data -Supporting Information file/Brats_17/Brats_t2_slice_77.png]

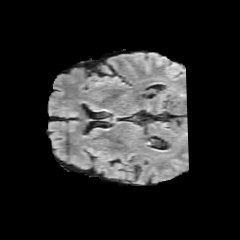

Supplement: S1 Data — (ZIP) [file pone.0325534.s001.zip › Partial data -Supporting Information file/Brats_18/Brats_flair_slice_77.png]

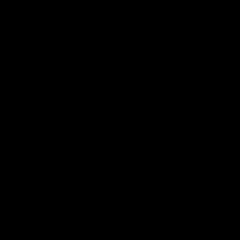

Supplement: S1 Data — (ZIP) [file pone.0325534.s001.zip › Partial data -Supporting Information file/Brats_18/Brats_seg_slice_77.png]

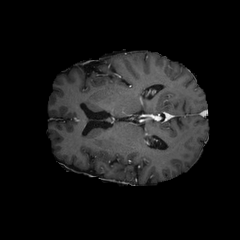

Supplement: S1 Data — (ZIP) [file pone.0325534.s001.zip › Partial data -Supporting Information file/Brats_18/Brats_t1ce_slice_77.png]

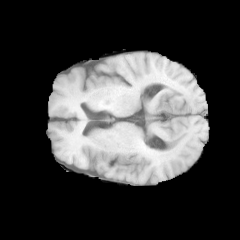

Supplement: S1 Data — (ZIP) [file pone.0325534.s001.zip › Partial data -Supporting Information file/Brats_18/Brats_t1_slice_77.png]

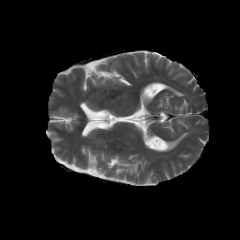

Supplement: S1 Data — (ZIP) [file pone.0325534.s001.zip › Partial data -Supporting Information file/Brats_18/Brats_t2_slice_77.png]

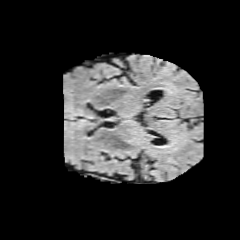

Supplement: S1 Data — (ZIP) [file pone.0325534.s001.zip › Partial data -Supporting Information file/Brats_19/Brats_flair_slice_77.png]

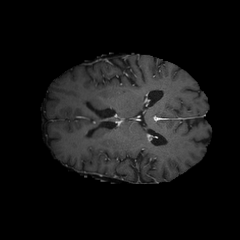

Supplement: S1 Data — (ZIP) [file pone.0325534.s001.zip › Partial data -Supporting Information file/Brats_19/Brats_t1ce_slice_77.png]

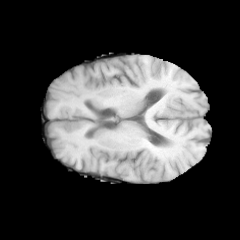

Supplement: S1 Data — (ZIP) [file pone.0325534.s001.zip › Partial data -Supporting Information file/Brats_19/Brats_t1_slice_77.png]

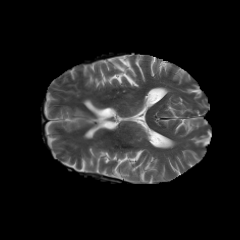

Supplement: S1 Data — (ZIP) [file pone.0325534.s001.zip › Partial data -Supporting Information file/Brats_19/Brats_t2_slice_77.png]

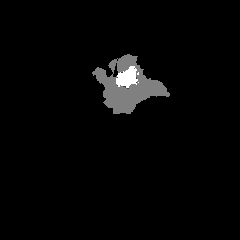

Supplement: S1 Data — (ZIP) [file pone.0325534.s001.zip › Partial data -Supporting Information file/Brats_2/Brats18_2013_3_1_seg_slice_77.png]

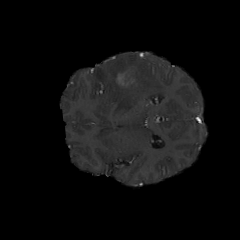

Supplement: S1 Data — (ZIP) [file pone.0325534.s001.zip › Partial data -Supporting Information file/Brats_2/Brats18_2013_3_1_t1ce_slice_77.png]

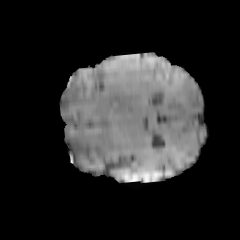

Supplement: S1 Data — (ZIP) [file pone.0325534.s001.zip › Partial data -Supporting Information file/Brats_2/Brats18_2013_3_1_t1_slice_77.png]

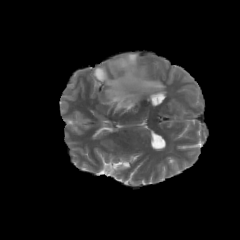

Supplement: S1 Data — (ZIP) [file pone.0325534.s001.zip › Partial data -Supporting Information file/Brats_2/Brats18_2013_3_1_t2_slice_77.png]

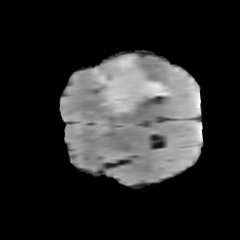

Supplement: S1 Data — (ZIP) [file pone.0325534.s001.zip › Partial data -Supporting Information file/Brats_2/Brats_flair_slice_77.png]

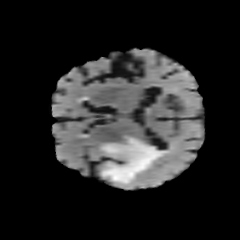

Supplement: S1 Data — (ZIP) [file pone.0325534.s001.zip › Partial data -Supporting Information file/Brats_20/Brats_flair_slice_77.png]

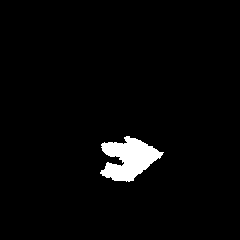

Supplement: S1 Data — (ZIP) [file pone.0325534.s001.zip › Partial data -Supporting Information file/Brats_20/Brats_seg_slice_77.png]

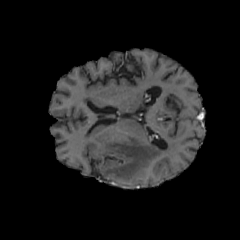

Supplement: S1 Data — (ZIP) [file pone.0325534.s001.zip › Partial data -Supporting Information file/Brats_20/Brats_t1ce_slice_77.png]

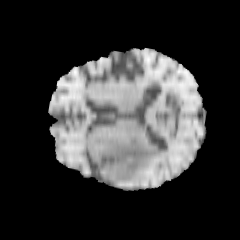

Supplement: S1 Data — (ZIP) [file pone.0325534.s001.zip › Partial data -Supporting Information file/Brats_20/Brats_t1_slice_77.png]

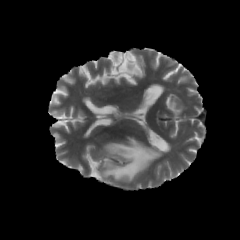

Supplement: S1 Data — (ZIP) [file pone.0325534.s001.zip › Partial data -Supporting Information file/Brats_20/Brats_t2_slice_77.png]

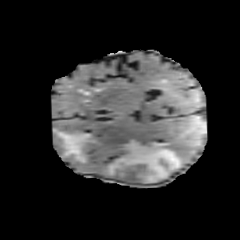

Supplement: S1 Data — (ZIP) [file pone.0325534.s001.zip › Partial data -Supporting Information file/Brats_3/Brats_flair_slice_77.png]

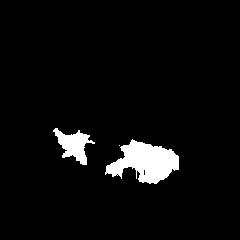

Supplement: S1 Data — (ZIP) [file pone.0325534.s001.zip › Partial data -Supporting Information file/Brats_3/Brats_seg_slice_77.png]

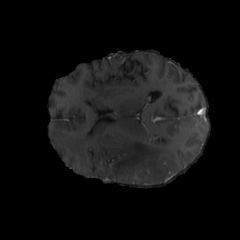

Supplement: S1 Data — (ZIP) [file pone.0325534.s001.zip › Partial data -Supporting Information file/Brats_3/Brats_t1ce_slice_77.png]

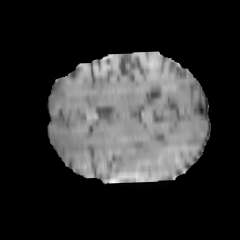

Supplement: S1 Data — (ZIP) [file pone.0325534.s001.zip › Partial data -Supporting Information file/Brats_3/Brats_t1_slice_77.png]

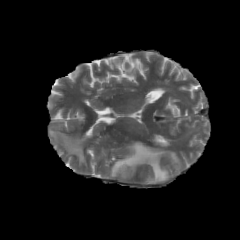

Supplement: S1 Data — (ZIP) [file pone.0325534.s001.zip › Partial data -Supporting Information file/Brats_3/Brats_t2_slice_77.png]

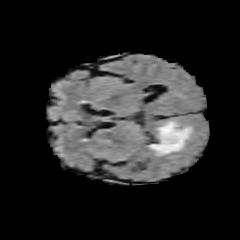

Supplement: S1 Data — (ZIP) [file pone.0325534.s001.zip › Partial data -Supporting Information file/Brats_4/Brats_flair_slice_77.png]

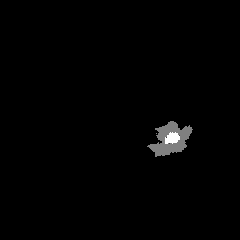

Supplement: S1 Data — (ZIP) [file pone.0325534.s001.zip › Partial data -Supporting Information file/Brats_4/Brats_seg_slice_77.png]

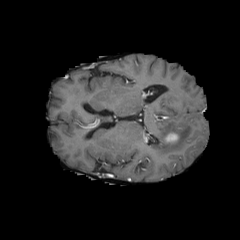

Supplement: S1 Data — (ZIP) [file pone.0325534.s001.zip › Partial data -Supporting Information file/Brats_4/Brats_t1ce_slice_77.png]

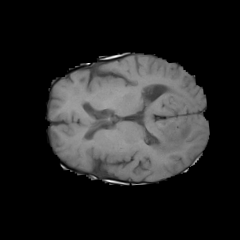

Supplement: S1 Data — (ZIP) [file pone.0325534.s001.zip › Partial data -Supporting Information file/Brats_4/Brats_t1_slice_77.png]

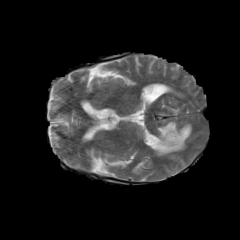

Supplement: S1 Data — (ZIP) [file pone.0325534.s001.zip › Partial data -Supporting Information file/Brats_4/Brats_t2_slice_77.png]

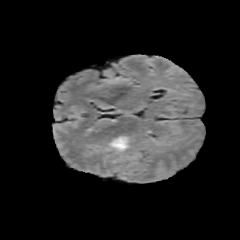

Supplement: S1 Data — (ZIP) [file pone.0325534.s001.zip › Partial data -Supporting Information file/Brats_5/Brats_flair_slice_77.png]

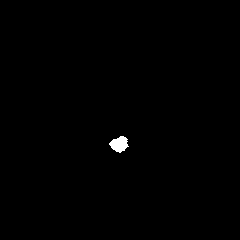

Supplement: S1 Data — (ZIP) [file pone.0325534.s001.zip › Partial data -Supporting Information file/Brats_5/Brats_seg_slice_77.png]

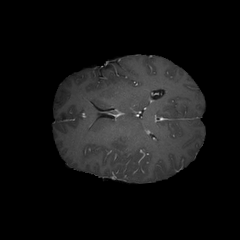

Supplement: S1 Data — (ZIP) [file pone.0325534.s001.zip › Partial data -Supporting Information file/Brats_5/Brats_t1ce_slice_77.png]

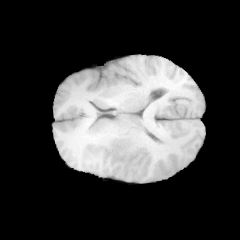

Supplement: S1 Data — (ZIP) [file pone.0325534.s001.zip › Partial data -Supporting Information file/Brats_5/Brats_t1_slice_77.png]

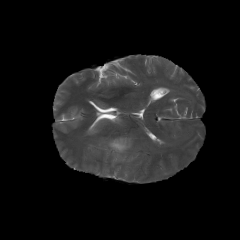

Supplement: S1 Data — (ZIP) [file pone.0325534.s001.zip › Partial data -Supporting Information file/Brats_5/Brats_t2_slice_77.png]

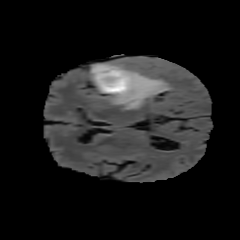

Supplement: S1 Data — (ZIP) [file pone.0325534.s001.zip › Partial data -Supporting Information file/Brats_6/Brats_flair_slice_77.png]

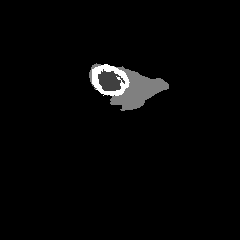

Supplement: S1 Data — (ZIP) [file pone.0325534.s001.zip › Partial data -Supporting Information file/Brats_6/Brats_seg_slice_77.png]

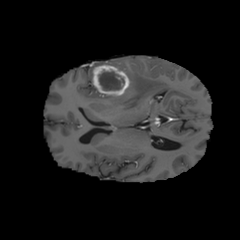

Supplement: S1 Data — (ZIP) [file pone.0325534.s001.zip › Partial data -Supporting Information file/Brats_6/Brats_t1ce_slice_77.png]

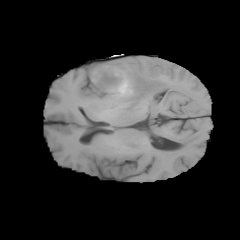

Supplement: S1 Data — (ZIP) [file pone.0325534.s001.zip › Partial data -Supporting Information file/Brats_6/Brats_t1_slice_77.png]

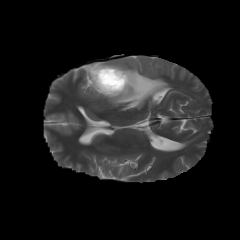

Supplement: S1 Data — (ZIP) [file pone.0325534.s001.zip › Partial data -Supporting Information file/Brats_6/Brats_t2_slice_77.png]

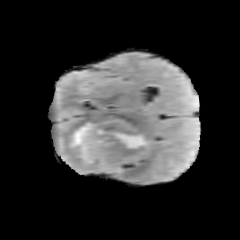

Supplement: S1 Data — (ZIP) [file pone.0325534.s001.zip › Partial data -Supporting Information file/Brats_7/Brats_flair_slice_77.png]

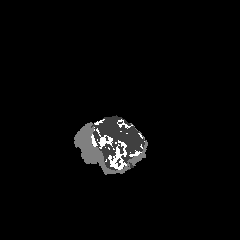

Supplement: S1 Data — (ZIP) [file pone.0325534.s001.zip › Partial data -Supporting Information file/Brats_7/Brats_seg_slice_77.png]

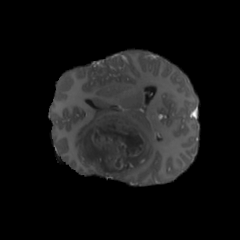

Supplement: S1 Data — (ZIP) [file pone.0325534.s001.zip › Partial data -Supporting Information file/Brats_7/Brats_t1ce_slice_77.png]

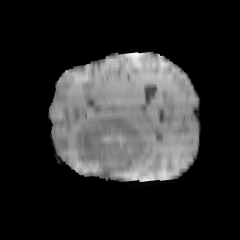

Supplement: S1 Data — (ZIP) [file pone.0325534.s001.zip › Partial data -Supporting Information file/Brats_7/Brats_t1_slice_77.png]

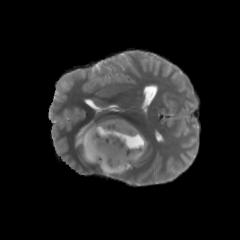

Supplement: S1 Data — (ZIP) [file pone.0325534.s001.zip › Partial data -Supporting Information file/Brats_7/Brats_t2_slice_77.png]

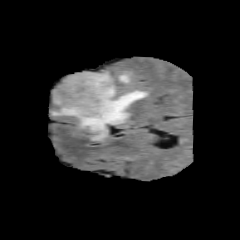

Supplement: S1 Data — (ZIP) [file pone.0325534.s001.zip › Partial data -Supporting Information file/Brats_8/Brats_flair_slice_77.png]

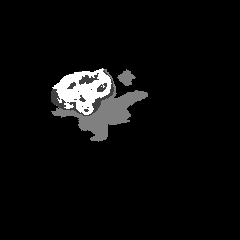

Supplement: S1 Data — (ZIP) [file pone.0325534.s001.zip › Partial data -Supporting Information file/Brats_8/Brats_seg_slice_77.png]

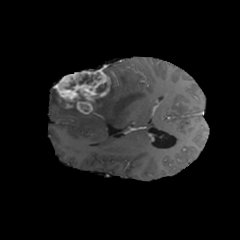

Supplement: S1 Data — (ZIP) [file pone.0325534.s001.zip › Partial data -Supporting Information file/Brats_8/Brats_t1ce_slice_77.png]

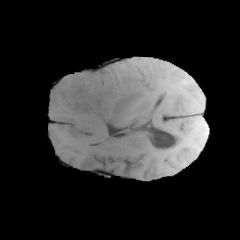

Supplement: S1 Data — (ZIP) [file pone.0325534.s001.zip › Partial data -Supporting Information file/Brats_8/Brats_t1_slice_77.png]

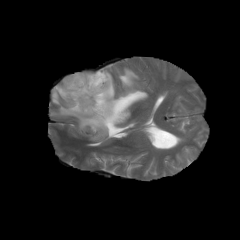

Supplement: S1 Data — (ZIP) [file pone.0325534.s001.zip › Partial data -Supporting Information file/Brats_8/Brats_t2_slice_77.png]

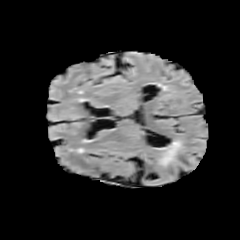

Supplement: S1 Data — (ZIP) [file pone.0325534.s001.zip › Partial data -Supporting Information file/Brats_9/Brats_flair_slice_77.png]

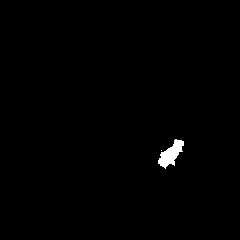

Supplement: S1 Data — (ZIP) [file pone.0325534.s001.zip › Partial data -Supporting Information file/Brats_9/Brats_seg_slice_77.png]

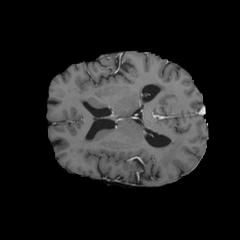

Supplement: S1 Data — (ZIP) [file pone.0325534.s001.zip › Partial data -Supporting Information file/Brats_9/Brats_t1ce_slice_77.png]

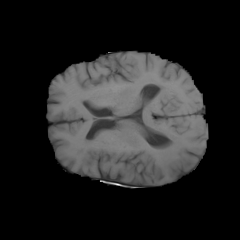

Supplement: S1 Data — (ZIP) [file pone.0325534.s001.zip › Partial data -Supporting Information file/Brats_9/Brats_t1_slice_77.png]

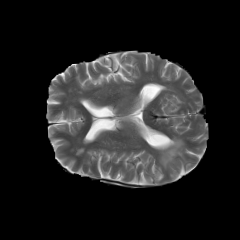

Supplement: S1 Data — (ZIP) [file pone.0325534.s001.zip › Partial data -Supporting Information file/Brats_9/Brats_t2_slice_77.png]
