# Supplementary material for: 3D-MRI brain glioma intelligent segmentation based on improved 3D U-net network
Source: PLoS One. 2025 Jun 13;20(6):e0325534. doi: 10.1371/journal.pone.0325534 (PMC12165427; doi:10.1371/journal.pone.0325534)
Supplement: Attachment — Submitted filename: Response to Reviewers.docx [file pone.0325534.s002.docx]

Dear Editors and Reviewers:

Thank you for your letter and for the reviewers’ comments concerning our manuscript entitled “3D-MRI Brain Glioma Intelligent Segmentation Based on Improved 3D U-Net Network” (ID: PONE-D-24-42605). Those comments are all valuable and very helpful for revising and improving our paper, as well as the important guiding significance to our researches. We have studied comments carefully and have made correction which we hope meet with approval.

Responds to the reviewer’s comments:

Reviewer #1:

Thank you very much for your professional review of our articles. In the revised version, your suggestions are highlighted in red. According to your suggestions, we have made a lot of modifications to the previous draft, and the specific modifications are as follows:

**The proposed 3D U-Net architecture seems to have some limitations: Firstly, novelty remains a concern; thus, the authors need to present the contributions clearly in the revised manuscript.**

**1. Due to the high number of parameters, it can easily overfit to the training data, especially if the dataset is small or not diverse enough.**
Reply: Based on the issues pointed out above, in order to solve the problem of imbalanced datasets with small or insufficient diversity, the article uses a loss function composed of Dice loss function and Focal loss function to train an improved 3D U-Net network model, in order to improve the model's generalization ability. The specific description begins with section 3.5 as follows:

This function comprises both the Dice loss and Focal loss functions. Train the improved 3D U-Net network model with the loss function as the optimization objective to comprehensively consider the accuracy of prediction results and class imbalance issues, thereby improving the model's generalization ability and ensuring the accuracy of 3D MRI segmentation of gliomas. The Dice loss function, which is the value obtained by subtracting the Dice coefficient from 1, transforms the voxel level labeling problem into a problem of minimizing inter class distance to learn the distribution of classes, and to some extent solves the problem of class imbalance; The Focal loss function changes the sample weights to make the model more inclined to focus on difficult to classify samples during training, in order to better learn voxels with classification errors.

**2. The model seems to be computationally intensive, requiring significant memory and processing power, which can be a barrier for some researchers or clinics. Is it possible to use the concept of parallel computation to overcome this? The authors could discuss this by referring the below papers:
“Real-time Automated Image Segmentation Technique for Cerebral Aneurysm on Reconfigurable System-On-Chip,” Journal of Computational Science, Elsevier, vol. 27, pp 35-45, 2018.
“Lattice-Boltzmann Interactive Blood Flow Simulation Pipeline,” International Journal of Computer Assisted Radiology and Surgery, Springer, vol.15, pp. 629-639, 2020.
“Zynq SoC based Acceleration of the Lattice Boltzmann Method,” Concurrency and Computation: Practice and Experience, Wiley, col. 31, issue 17, 2019.
"Heterogeneous System-on-Chip based Lattice- Boltzmann Visual Simulation System,” Systems Journal, IEEE, vol. 14, no. 2, pp. 1592-1601, 2020**
Reply: Based on the suggestions, a search was conducted on the provided literature, and in terms of processing efficiency, corresponding descriptions were made in the article according to the concept of parallel computing, as follows:

And to cope with the large amount of memory and processing power required for computationally intensive models, the concept of parallel computing is used to overcome this problem. The hollow space pyramid pooling structure introduced in the 3D U-Net network and the network structure of the multi-scale fusion attention module are run separately on different GPUs, and data transmission is carried out through high-speed communication links between the hollow space pyramid pooling structure and the network structure of the multi-scale fusion attention module to ensure communication and data synchronization between different GPUs. And the tasks in the multi-scale fusion attention module can also be decomposed into smaller tasks, that is, the calculation of different scale features can be performed in parallel on different computing units, and then the fusion operation can be performed. Based on this, to some extent, the problem of high memory and processing power requirements brought by the model can be overcome, and overall efficiency can be improved.

**3. In cases where the glioma regions are much smaller compared to healthy tissue, the model may have difficulty learning the minority class effectively, leading to poor segmentation performance.
The performance may be adversely affected by noise and artifacts in the MRI images, which are common in clinical settings. The authors need to discuss if a pre-processing using stocastic resonance theory can be of help. The authors could refer the bew studies while discussing this: "Development of a Cerebral Aneurysm Segmentation Method to Prevent Sentinel Hemorrhage," Network Modeling Analysis in Health Informatics and Bioinformatics, Springer, vol. 12, no. 18, pp. 1-14, 2023.
"Toward Computing Cross-Modality Symmetric Non-Rigid Medical Image Registration," IEEE Access, vol. 10, pp. 24528-24539, 2022,
"Moving Object Tracking in Clinical Scenarios: Application to Cardiac Surgery and Cerebral Aneurysm Clipping,” International Journal of Computer Assisted Radiology and Surgery, Springer, vol. 14, no. 12, pp. 2165-2176, 2019.
“A PCA based Approach for Brain Aneurysm Segmentation,” Journal of Multi Dimensional Systems and Signal Processing, Springer, vol. 29, pp. 257-277, 2018.**
Reply: Based on the suggestions, the provided literature has been referenced, and in section 2.2, preprocessing based on stochastic resonance theory has been added to avoid the adverse effects of noise on subsequent segmentation. The specific supplementary content is as follows:

Based on the above corrections, in order to further avoid the impact of noise on the subsequent segmentation performance of MRI images, we now proceed with processing according to the theory of stochastic resonance. Random resonance is a nonlinear phenomenon, and under certain conditions, the addition of noise can enhance the detection ability of weak signals. In MRI images, although noise is often considered interference, it is possible to transform noise into a factor that helps improve image quality by utilizing stochastic resonance theory. From the perspective of signal processing, the signal in MRI images can be seen as a mixed signal consisting of target tissue information (useful signal) and noise and artifacts (interference signal). The theory of stochastic resonance can enhance useful signals in the presence of noise by adjusting certain parameters of the system. The specific implementation process is as follows:

Firstly, a method based on local statistical information is used to estimate the noise level in MRI images, as shown below:

$D=k\sigma^{2}$（5）

In the formula, $D$ represents the noise intensity; $k$ is the adjustment coefficient, which can take values between 0.1-10; $\sigma^{2}$ is the noise variance.

Secondly, the modified and normalized data mentioned above is used as the amplitude $A$ of the input MRI image signal, which is then fed into a bistable system. The dynamic equation can be expressed as:

$\frac{dx}{dt}=x-x^{3}+A\sin\left(\omega t\right)+\sqrt{2D}\xi\left(t\right)$（6）

In the formula, $x$ is the state variable of the system; $\omega$ is the angular frequency of the input signal, with an initial value of 1, and then adjusted according to the processing result; $\xi\left(t\right)$ is Gaussian white noise.

Then, numerical methods are used to solve the dynamic equations of the bistable system, and the output signal after stochastic resonance processing is obtained. For the grayscale value of each pixel, the value of the system's state variable $x$ is gradually calculated according to a set time step (such as $\Delta t=0.01$). After a certain period of time (such as $T=100$ time units), the stable value of $x$ is taken as the processed grayscale value of the pixel, and the corresponding output signal is obtained.

Finally, the one-dimensional signal processed by stochastic resonance is converted back into a two-dimensional image. Evaluate the processed image using the Peak Signal to Noise Ratio (PSNR) image quality assessment metric. If the evaluation indicators do not improve, the parameters of the stochastic resonance system (such as noise intensity $D$, angular frequency $\omega$) can be adjusted and reprocessed until satisfactory image quality improvement is achieved.

**4. The authors are encourage to include the potential limitations of the paper.**
Reply: According to the suggestion, a description of the limitations of the proposed method has been added to the discussion section in Section 5, as follows:

Although the proposed method overcomes the problem of large memory and processing power required for computationally intensive models based on the concept of parallel computing, there are still issues of introducing additional overhead and increasing computing resources.

**5. Please discuss the present computational complexity of the model.**

Reply: According to the suggestion, an analysis on computational complexity has been added in Section 4, as follows:

To further analyze the computational complexity of the proposed method, the methods in references [11], [12], [13], [14], and [15] were used as comparative methods, and time was used to measure the computational complexity. The time complexity can reflect the upward trend of algorithm execution time with increasing input size. Therefore, based on the BraTS2018 public training dataset, 210 samples of advanced glioblastoma (HGG) were used for testing. Six methods were used to segment samples with gradually increasing data size from 10 to 210, measured by linear time complexity. The results are shown in Figure 11.

Figure 11 Linear time complexity results

According to the results in Figure 11, using six methods for access control, the linear time complexity results show an upward trend. As the data size increases, the execution time of the methods also increases linearly. However, upon comparison, it can be found that using the proposed method for segmentation results in a lower trend in linear time complexity compared to the other five literature methods. This indicates that the proposed method requires less time and is more efficient when processing data of the same scale. This advantage may stem from the proposed method being more optimized in design, utilizing parallel processing techniques to improve efficiency and resulting in lower computational complexity.

Reviewer #2:

We sincerely thank reviewers for their valuable comments, which we use to improve the quality of our manuscripts. The reviewer's comments are listed below in bold, with specific questions numbered. Our replies are given in green text.

**1. "hyperparametric loss function by combining the Dice loss and Focal loss functions" This is a well known combo loss in the literature.**
Reply: The pointed out issue has been checked in the article, and its innovation does not lie in combining Dice loss and focus loss functions. Instead, a hollow space pyramid pooling structure and a multi-scale fusion attention module are introduced to improve the processing of the 3D U-Net network. While accurately capturing contextual information of different scales in glioma MRI, the detailed information of glioma is enhanced, and useless background information is suppressed to improve segmentation performance. Based on this, in order to improve the segmentation accuracy and generalization ability of the model, a loss function combining Dice loss function and Focal loss function is used to train the improved model, enhance its learning ability for difficult to distinguish samples, and further improve segmentation performance.

**2. Use of pyramid scene parsing and ASPP has been popular for medical image segementation:**
**a. A lightweight neural network with multiscale feature enhancement for liver CT segmentation
b. Dense-PSP-UNet: A neural network for fast inference liver ultrasound segmentation**
Reply: Based on the pointed out issues, the proposed method introduces the advantages of the hollow space pyramid pooling structure and multi-scale fusion attention module into the 3D U-Net network to improve the 3D U-Net network. The two structures complement each other, making significant progress in capturing multi-scale information, enhancing feature extraction ability, and improving segmentation accuracy. This improves the effectiveness of the 3D U-Net network in 3D MRI glioma segmentation and provides strong support for accurate diagnosis and treatment of gliomas.

**3. Authors claim that registration is part of their method. However, no details of registration have been shared in the the methods section.**
Reply: Based on the identified issues, corresponding implementation process content has been added in section 2.1 of the article, as follows:

During the registration process, the deformation parameters of the input image were continuously adjusted to minimize the difference between them and the reference image. The specific implementation process is described as follows:

During the registration process, the mean square error (MSE) is used to measure the similarity between the input image and the reference image, with the goal of minimizing this MSE value. The mean square error is expressed as follows:

$MSE=\frac{1}{N}\sum_{i=1}^{N} \left(I_{1}\left(i\right)-I_{2}\left(i\right)\right)^{2}$（1）

In the formula, $I_{1}\left(i\right)$ is the input image; $I_{2}\left(i\right)$ is the reference image; $N$ is the total number of pixels.

Registration continuously adjusts the deformation parameters of the input image to minimize the difference between it and the reference image. This deformation parameter is usually represented by a displacement vector field, namely deformation field $d\left(x,y,z\right)$, which describes the deformation mapping from the input image to the reference image, that is, each pixel (or voxel) in each direction has a corresponding displacement vector. $d\left(x,y,z\right)$ represents as follows:

$d\left(x,y,z\right)=mathbf\left(d_{x}\left(x,y,z\right),d_{y}\left(x,y,z\right),d_{z}\left(x,y,z\right)\right)$（2）

To prevent excessive deformation, a regularization term is added during the registration process to constrain the smoothness of the deformation field. The regularization term is represented as follows:

$E_{reg}=\int\left\|\nabla d\left(x,y,z\right)\right\|^{2}dxdydz$（3）

In the equation, $\nabla d\left(x,y,z\right)$ is the gradient of the displacement vector; $\left\|\nabla d\left(x,y,z\right)\right\|$ is the modulus of the gradient vector.

Then, through iterative optimization, continuously adjust the deformation parameters. In each iteration, the similarity measure and regularization term will be calculated based on the current deformation parameters, and the deformation parameters will be updated using gradient descent until the MSE value of the similarity measure reaches the minimum and satisfies the constraint of formula (3), ending the iteration and completing the registration.

**4. What's the distinction between MFAB and fusion module? Network figure has to be redesigned and explanation should be made clear.**

Reply: The difference between MFAB and fusion module is that MFAB emphasizes channel attention mechanism, which enhances or suppresses feature maps by learning the importance of each feature map channel. However, typical fusion modules do not have this explicit channel attention mechanism.

And MFAB pays special attention to the integration of multi-scale features, which helps capture various information in images. And some fusion modules may only focus on feature integration at specific scales.

And based on the suggestions, the structure of the multi-scale fusion attention module was redrawn.

**5. Please dont exaggerate loss as super parameter or hyperparametric.**
Reply: According to the suggestion, unify the hyperparameter loss function into a loss function.

**6. training loss is not interesting why not share validation and test losses for Tables 1 and 2.**
Reply: Table 2 shows the robustness results of each model to interference on the test dataset. Therefore, according to the suggestion, Table 1 should be changed to the loss on the validation and test sets, as follows:

The efficacy of the brain glioma segmentation model significantly influences its segmentation accuracy. Utilizing the 3D U-Net network as a foundational structure, this paper introduces the ASPP network and a multi-scale fusion attention module, MFAB, to conduct ablation studies. Three brain glioma segmentation network models were developed: the 3D U-Net network model, the 3D U-Net+ASPP network model, and the enhanced 3D U-Net network model. These models were trained using 118 samples from the BraTS2018 dataset, and by comparing the loss changes of each network model in the validation dataset and the test dataset, the performance of each network model was studied. The experimental results are shown in Table 1.

**Table 1 Change analysis of loss function before and after improvement of glioma segmentation model**

| Data set | Training frequency | Training losses | | |
| --- | --- | --- | --- | --- |
|  |  | 3D U-Net model | 3D U-Ne+ASPP model | Improved 3D U-Net Model |
| Validate the dataset | 0 | 0.77 | 0.50 | 0.58 |
|  | 80 | 0.58 | 0.39 | 0.18 |
|  | 160 | 0.50 | 0.35 | 0.10 |
|  | 240 | 0.54 | 0.40 | 0.10 |
|  | 320 | 0.59 | 0.43 | 0.10 |
| Test dataset | 0 | 0.78 | 0.51 | 0.58 |
|  | 80 | 0.59 | 0.41 | 0.18 |
|  | 160 | 0.51 | 0.41 | 0.10 |
|  | 240 | 0.50 | 0.36 | 0.10 |
|  | 320 | 0.50 | 0.36 | 0.10 |

According to Table 1, there are significant differences in the performance of the three models between the validation dataset and the test dataset. Among them, in the validation dataset, the 3D U-Net network model and the 3D U-Net+ASPP network model showed overfitting with increasing training times. The improved 3D U-Net network model is stable and performs better than the 3D U-Net network model and the 3D U-Net+ASPP network model in both the validation and testing datasets. In the test dataset, as the number of training iterations increases, the training loss of each glioma segmentation network model shows a decreasing trend, and the final region stabilizes. The improved 3D U-Net network model showed a rapid and significant decrease in training loss during the first 80 model training sessions. After further increasing the number of training sessions, the model's training loss showed a lower degree of change. When the number of training sessions reached 160, the model's training loss began to stabilize, with a final training loss of 0.1; During the training process of the 3D U-Net network model, the training loss was significantly reduced in the first 80 iterations. Before reaching 260 training iterations, the loss value showed a slow downward trend. After 260 training iterations, the model gradually reached a convergence state, and its training loss was much higher than that of the improved glioma segmentation model, with a training loss of 0.5. The training loss of the 3D U-Net+ASPP network model is lower than that of the original 3D U-Net network model due to the introduction of ASPP structure. After 240 training sessions, it stabilizes at 0.36. The experimental results show that, based on the 3D U-Net network model, the introduction of ASPP structure and multi-scale fusion attention module MFAB can effectively learn the features of brain glioma MRI images from training samples, eliminate the influence of irrelevant information on segmentation results, and improve the segmentation effect of brain gliomas. The improved 3D U-Net network model has outstanding performance.

**7. HD distance as a metrics is skipped in the results section.**
Reply: According to the suggestions, the analysis of HD distance indicators has been checked, and Figure 10 shows the results of HD distance indicators, as follows:

On the basis of the above, in order to quantify the segmentation performance of each method, the HD distance index is now used to measure the segmentation results of each method on the 10 FLAIR modal image samples mentioned above. The results are shown in Figure 10.

Figure 10 HD distance results

According to the results in Figure 10, using the method proposed in this paper to segment 10 FLAIR modal image samples, the HD distance index remained above 0.98. However, after using the method in reference [12] for segmentation, the highest HD distance index among all FLAIR modal image samples could only reach 0.92. Therefore, comparing the HD distance index results of the two methods, it can be concluded that the difference between the predicted segmentation boundary and the true boundary in this paper's method is small, which can better capture the contour of glioma and has high segmentation accuracy. This is because the method proposed in this article uses 3D U-Net as the basic structure, introduces a hollow space pyramid pooling structure and a multi-scale fusion attention module to improve the processing of the 3D U-Net network, achieving accurate capture of contextual information at different scales in brain glioma MRI while enhancing the detailed information of the glioma and suppressing useless background information, thereby improving the performance and effectiveness of segmentation and achieving accurate segmentation of glioma regions.

**8. Lack of comparison with literature. At least 5-7 methods should be implemented and compared with the proposed method.**
Reply: According to the suggestion, a comparative analysis between the 5 comparison methods and the proposed method has been added in Section 4, as follows:

To further analyze the computational complexity of the proposed method, the methods in references [11], [12], [13], [14], and [15] were used as comparative methods, and time was used to measure the computational complexity. The time complexity can reflect the upward trend of algorithm execution time with increasing input size. Therefore, based on the BraTS2018 public training dataset, 210 samples of advanced glioblastoma (HGG) were used for testing. Six methods were used to segment samples with gradually increasing data size from 10 to 210, measured by linear time complexity. The results are shown in Figure 11.

Figure 11 Linear time complexity results

According to the results in Figure 11, using six methods for access control, the linear time complexity results show an upward trend. As the data size increases, the execution time of the methods also increases linearly. However, upon comparison, it can be found that using the proposed method for segmentation results in a lower trend in linear time complexity compared to the other five literature methods. This indicates that the proposed method requires less time and is more efficient when processing data of the same scale. This advantage may stem from the proposed method being more optimized in design, utilizing parallel processing techniques to improve efficiency and resulting in lower computational complexity.

**9. Discussion is missing from the work.**
Reply: According to the suggestion, a discussion section 5 has been added to the text, as follows:

The innovative contribution of this study lies in improving the ability of the model to distinguish challenging samples. By introducing a hollow space pyramid pooling structure and a multi-scale fusion attention module, the 3D U-Net network is improved to accurately capture contextual information of different scales in glioma MRI, while enhancing the detailed information of glioma and suppressing useless background information. The Dice loss function and Focal loss function are combined to train the improved 3D U-Net network model, thereby optimizing segmentation accuracy and generalization ability, ultimately improving the accuracy and reliability of diagnosis, and providing a scientific basis for clinical diagnosis and treatment. The test results show that the training loss of the proposed method is only 0.1, with DSC, Recall, and Precision values of 0.7512, 0.7064, and 0.77451, respectively; In whole tumor (WT) segmentation, the Dice similarity coefficient (DSC), recall rate, and accuracy scores were 0.9168, 0.9426, and 0.9375, respectively; For the segmentation of core tumors (TC), these scores are 0.8954, 0.9014, and 0.9369, respectively; In enhanced tumor (ET) segmentation, the DSC, Recall, and Precision values of this method are 0.8674, 0.9045, and 0.9011, respectively; And the HD distance index remains above 0.98, with low time complexity. This indicates that the introduction of hollow space pyramid pool structure and multi-scale fusion attention module to improve the processing of 3D U-Net network, and based on the concept of parallel computing to overcome the problem of requiring a large amount of memory and processing power for the model to be computationally intensive, effectively enhances the 3D U-Net segmentation model and exhibits excellent performance.

Although the proposed method overcomes the problem of large memory and processing power required for computationally intensive models based on the concept of parallel computing, there are still issues of introducing additional overhead and increasing computing resources. Therefore, in the future, research will be conducted towards optimizing model structure and computational efficiency to explore more efficient network architectures, such as lightweight 3D convolutional neural networks, to reduce the consumption of computing resources and memory.
**10. Following works should be cited to encourage the use of DL/CNN/UNet for brain tumor segmentation:
a. Towards developing a lightweight neural network for liver CT segmentation.
b. Neural network-based fast liver ultrasound image segmentation
c. Unveiling the future of breast cancer assessment: a critical review on generative adversarial networks in elastography ultrasound
d. Advancements in Deep Learning for B-Mode Ultrasound Segmentation: A Comprehensive Review
e. Estimating age and gender from electrocardiogram signals: A comprehensive review of the past decade
f. Practical utility of liver segmentation methods in clinical surgeries and interventions**

Reply: According to the suggestion, the literature "Neural network-based fast liver ultrasound image segmentation" and "Advances in Deep Learning for B-mode Ultrasound Segmentation: A Comprehensive Review" were cited in the text, and the subsequent literature list was updated.
