# Supplementary material for: 3D-MRI brain glioma intelligent segmentation based on improved 3D U-net network
Source: PLoS One. 2025 Jun 13;20(6):e0325534. doi: 10.1371/journal.pone.0325534 (PMC12165427; doi:10.1371/journal.pone.0325534)
Supplement: Attachment — Submitted filename: Response_to_Reviewers_auresp_2.docx [file pone.0325534.s003.docx]

Dear Editors and Reviewers:

Thank you for your letter and for the reviewers’ comments concerning our manuscript entitled “3D-MRI Brain Glioma Intelligent Segmentation Based on Improved 3D U-Net Network” (ID: PONE-D-24-42605R1). Those comments are all valuable and very helpful for revising and improving our paper, as well as the important guiding significance to our researches. We have studied comments carefully and have made correction which we hope meet with approval.

Responds to the reviewer’s comments:

**Reviewer #3:**

Thank you very much for your professional review of our articles. According to your suggestions, we have made a lot of modifications to the previous draft, and the specific modifications are as follows:

**This paper resembles a technical report rather than a scientific research article. While the performance results are notable, the following concerns must be addressed before it can be considered for publication.
1.Writing and Grammar:
The overall writing of the manuscript requires improvement. For example, phrases like "Validate the dataset" and "Test dataset" on page 14 are unclear. There are grammar errors and clarity issues throughout the manuscript. Tools such as ChatGPT or other AI-based writing assistants can help enhance the writing quality. Ensure that every sentence in the manuscript is reviewed for grammatical accuracy and clarity.**
Reply: Based on the suggestions made, the expressions of phrases such as "validation dataset" and "test dataset" on page 14 of the article have been adjusted to make them clearer. The adjusted version is as follows:

According to Table 1, there is a significant difference in the performance of the 3D U-Net network model and the 3D U-Net+ASPP network model between the validation dataset and the test dataset, indicating the presence of overfitting. Among them, in the test dataset, the 3D U-Net network model and the 3D U-Net+ASPP network model showed a decreasing trend with increasing training times. Compared to the losses of the three models, the improved 3D U-Net network model has lower losses than the 3D U-Net network model and the 3D U-Net+ASPP network model. In the test dataset, as the number of iterations increases, the loss of each glioma segmentation network model shows a decreasing trend and ultimately remains stable.

And the grammar of the entire text was checked, and the problematic areas were modified. For example, on page 4, the sentence was changed to:

Then, through iterative optimization, the deformation parameters are continuously adjusted.

The paragraph on page 6 should be revised as follows:

To improve segmentation performance, this paper enhances the 3D U-Net network by integrating Atrous Spatial Pyramid Pooling (ASPP) and a multi-scale fusion attention module (MFAB). The ASPP module captures contextual information across multiple scales in 3D images, while the MFAB module focuses on identifying salient features of brain glioma regions in 3D-MRI scans and suppressing irrelevant areas. Furthermore, a combined loss function, which incorporates both Dice and Focal loss functions, is introduced to further enhance the segmentation accuracy of 3D-MRI brain glioma.
**2.State-of-the-Art (SOTA) Comparison:
To demonstrate that your method is truly state-of-the-art, it should be compared with the latest models on up-to-date datasets, such as those from the BraTS 2023 challenge. Since most SOTA methods no longer compete on older datasets, references to BraTS 2018 are largely outdated. Additionally, the baseline model used in the paper, U-Net with ASPP, is nearly 9 years old and does not reflect the latest advancements in the field.**
Reply: Based on the issues pointed out above, the dataset used in the article can be replaced with BraTS 2023.

In order to compare the baseline model U-Net with ASPP with the proposed model, ablation experiments were conducted to verify the improvement effect of the proposed model.

**3.Emphasis on Novelty:
To highlight the novelty of this paper, focus on identifying existing challenges and proposing innovative solutions, rather than combining or stacking existing methods. A clear distinction of how your approach addresses unmet needs in the field will strengthen the paper.**
Reply: Based on the suggestions made, the description at the end of the introduction on page 3 has been adjusted to further highlight the innovation of the proposed method. The revised version is as follows:

this paper introduces an enhanced 3D U-Net network for intelligent segmentation of gliomas in 3D MRI scans. Traditional 3D U-Net networks struggle to capture contextual information of different scales simultaneously when processing brain glioma MRI data, resulting in poor segmentation performance in tumor boundaries and detail areas. This article effectively solves this problem by introducing the Hollow Space Pyramid Pooling Structure (ASPP). ASPP can capture multi-scale contextual information without reducing resolution through multi-scale dilated convolution operations, thereby achieving accurate segmentation of glioma regions. To further enhance the detailed information of gliomas and suppress useless background information, a multi-scale fusion attention module is introduced. This module significantly improves the sensitivity of the network to tumor details through multi-scale feature fusion and attention mechanism, while effectively reducing the interference of background noise, making the segmentation results more accurate and reliable. To tackle the issue of class imbalance and enhance the learning of misclassified voxels, we devise a loss function by combining the Dice loss and Focal loss functions. This approach not only preserves segmentation accuracy but also improves the model's ability to learn from challenging samples, leading to enhanced segmentation performance, precision, and generalization.

**4.Background Information:
Commonly known background concepts, such as the original U-Net architecture, ASPP diagrams, multi-scale fusion attention modules, and formulas for Dice/recall/precision, should be abbreviated or summarized concisely to avoid redundancy.**
Reply: Based on the above suggestions, corresponding abbreviations have been made to the background concept description pointed out, for example, Figure 4 has been modified to:

Fig 4. Basic structure of MFAB module

On page 8, the sentence description is adjusted to:

To enhance segmentation, this paper integrates a MFAB into the 3D U-Net network.

**5.Overfitting and Data Inclusion:
Changing the loss function alone does not adequately address the overfitting problem. A more effective approach would be to incorporate additional public datasets into the training process. This would improve the model's generalizability and robustness.**
Reply: According to the suggestion, the description of adding additional public datasets to solve the overfitting problem has been added to the dataset description on page 13. As follows:

Using the BraTS2023 public training dataset as the research object, 60 glioma samples were randomly selected as the test dataset, including 15 cases of LGG and 45 cases of HGG. 80% of the remaining samples were used as the training dataset and 20% as the validation dataset. Among them, the number of samples in the training dataset is 180, including 55 cases of LGG and 125 cases of HGG; The sample size in the validation dataset is 45 cases, including 18 cases of LGG and 27 cases of HGG. To solve the problem of overfitting and improve the generalization ability and robustness of the model, the BraTS2023 public training dataset was used as the basis, and the BraTS2021 dataset was introduced for training, which also contains a large number of annotated brain glioma MRI images. Thus, 300 samples from BraTS2021 were introduced, and the two training datasets were merged to expand to 480 samples.

**6.Test Set Usage (Page 14, Lines 454–455):
The statement, "In the test dataset, as the number of training iterations increases, the training loss of each glioma segmentation network model shows a decreasing trend, and the final region stabilizes," raises serious concerns. Did you inadvertently include the test set during training? This practice is entirely unacceptable, as it compromises the validity of your results. If the improvement in Dice score is derived from using the test set during training, this constitutes a form of cheating. The test set must remain completely unseen during training and should only be used for final evaluation.**
Reply: Based on the identified issues, adjustments have been made to the description of the analysis results on page 14 to ensure their validity. The revised version is presented as follows:

According to Table 1, there is a significant difference in the performance of the 3D U-Net network model and the 3D U-Net+ASPP network model between the validation dataset and the test dataset, indicating the presence of overfitting. Among them, in the test dataset, the 3D U-Net network model and the 3D U-Net+ASPP network model showed a decreasing trend with increasing training times. Compared to the losses of the three models, the improved 3D U-Net network model has lower losses than the 3D U-Net network model and the 3D U-Net+ASPP network model. In the test dataset, as the number of iterations increases, the loss of each glioma segmentation network model shows a decreasing trend and ultimately remains stable. The improved 3D U-Net network model showed a rapid decrease in loss during the first 80 iterations, with a significant decrease. After increasing the number of iterations, the model loss exhibited a lower degree of change. When the number of iterations reached 160, the model loss began to stabilize, with a final loss of 0.11, which was not significantly different from the representation in the validation dataset; During the iteration process, the loss of the 3D U-Net network model significantly decreased in the first 80 iterations. Before reaching 260 iterations, the loss value showed a slow downward trend, and the model slowly reached a convergence state after 260 iterations. Its loss was much higher than that of the improved glioma segmentation model, with a loss of 0.50, but there was a significant gap compared to its performance in the validation dataset. The loss of the 3D U-Net+ASPP network model is lower than that of the original 3D U-Net network model due to the introduction of ASPP structure. After 240 iterations, it stabilizes at 0.36, which is significantly different from the performance page in the validation dataset. The experimental results show that, based on the 3D U-Net network model, the introduction of ASPP structure and multi-scale fusion attention module MFAB can effectively learn the features of glioma MRI images from training samples, eliminate the influence of irrelevant information on segmentation results, and introduce additional datasets during training to improve the generalization ability and robustness of the model, thereby enhancing the glioma segmentation effect. This indicates that the improved 3D U-Net network model has outstanding performance.

**7.Validation vs. Test Set Results (Table 1):
In Table 1, the loss values for the validation set and the test set are shown to be exactly the same. This is highly unusual and warrants a thorough review. Please double-check your experimental setup and ensure there are no errors in your data partitioning or evaluation process.**

Reply: Based on the issues pointed out, the values in Table 1 have been revised as follows:

**Table 1. Change analysis of loss function before and after improvement of glioma segmentation model**

| Data set | Training frequency | Losses | | |
| --- | --- | --- | --- | --- |
|  |  | 3D U-Net model | 3D U-Ne+ASPP model | Improved 3D U-Net Model |
| Validate the dataset | 0 | 0.77 | 0.58 | 0.50 |
|  | 80 | 0.58 | 0.39 | 0.18 |
|  | 160 | 0.50 | 0.35 | 0.10 |
|  | 240 | 0.54 | 0.40 | 0.10 |
|  | 320 | 0.59 | 0.43 | 0.10 |
| Test dataset | 0 | 0.78 | 0.65 | 0.52 |
|  | 80 | 0.59 | 0.41 | 0.19 |
|  | 160 | 0.51 | 0.41 | 0.11 |
|  | 240 | 0.50 | 0.36 | 0.11 |
|  | 320 | 0.50 | 0.36 | 0.11 |

**Reviewer #4:**

We sincerely thank reviewers for their valuable comments, which we use to improve the quality of our manuscripts. The reviewer's comments are listed below in bold, with specific questions numbered.

**Expand Comparative Experiments:
Include comparisons with recent high-performing glioma segmentation methods (e.g., those utilizing Transformers or advanced 3D architectures) to comprehensively evaluate the proposed method’s competitiveness.**

Reply: Based on the suggestions made, the content of Part 3 has been checked. Regarding the segmentation effect, a comparative analysis with 3D architecture methods is provided in the description on page 18, as follows:

To ensure the applicability of the proposed model, the BratS2020 dataset was used as the test set, and 10 samples of 3D MRI glioma cases were selected. FLAIR modal images have good contrast for observing the boundaries of gliomas and surrounding normal brain tissue. Gliomas often have irregular boundaries and blurry areas between them and surrounding normal brain tissue. FLAIR images can help doctors observe these boundaries more clearly, thereby aiding in accurate segmentation of lesions. Therefore, FLAIR modality MRI images were selected for testing. Images of the 10 samples are presented in Fig 6. To assess the segmentation effectiveness of the proposed method, the highly efficient three-dimensional residual neural network segmentation approach from literature[12] was utilized as a comparative method. This approach segmented FLAIR MRI images of the 3D-MRI brain glioma samples alongside the proposed method, with results compared to manual annotations. Differences in brain glioma segmentation outcomes were analyzed, with experimental results showcased in Figures 7, 8, and 9.

**Fig 6. Original 3D-MRI brain glioma case sample data (a) The 1st 3D-MRI glioma sample (b) The 2nd case of 3D-MRI glioma (c) The 3rd case of 3D-MRI glioma (d) The 4th case of 3D-MRI glioma (e) The 5th case of 3D-MRI glioma (f) The 6th case of 3D-MRI glioma**

**Fig 7. Sample labeling results of 3D-MRI glioma cases (a) The 1st 3D-MRI glioma sample (b) The 2nd case of 3D-MRI glioma (c) The 3rd case of 3D-MRI glioma (d) The 4th case of 3D-MRI glioma (e) The 5th case of 3D-MRI glioma (f) The 6th case of 3D-MRI glioma**

**Fig 8. The segmentation results of 3D-MRI glioma case samples using the method described in this article (a) The 1st 3D-MRI glioma sample (b) The 2nd case of 3D-MRI glioma (c) The 3rd case of 3D-MRI glioma (d) The 4th case of 3D-MRI glioma (e) The 5th case of 3D-MRI glioma (f) The 6th case of 3D-MRI glioma**

**Fig 9. Sample segmentation results of 3D-MRI glioma cases using comparative methods (a) The 1st 3D-MRI glioma sample (b) The 2nd case of 3D-MRI glioma (c) The 3rd case of 3D-MRI glioma (d) The 4th case of 3D-MRI glioma (e) The 5th case of 3D-MRI glioma (f) The 6th case of 3D-MRI glioma**

From the analysis of Fig 7, 8 and 9, it is evident that FLAIR mode MRI segmentation in this study accurately identifies the complete tumor region WT, core tumor region TC, and enhanced tumor region ET in the MRI samples. The enhanced tumor region ET is marked in red, while the core tumor region TC is indicated by a combination of red and blue. The complete tumor region WT encompasses areas labeled in red, blue, and green. In this study's MRI samples, the brain glioma regions for the 1st, 3rd, 7th, and 9th cases were located in the left hemisphere; for the 2nd case, above the right hemisphere; for the 4th, 5th, 8th, and 10th cases, in the right hemisphere; and for the 6th case, in the lower part of the right hemisphere. Notably, the enhanced tumor area in the 10 examples was relatively smaller than other tumor areas, making it susceptible to misclassification. Comparing the FLAIR modal MRI segmentation results of the current method with those in reference[12] and the manually annotated results in Fig 7, the enhanced tumor regions segmented by this study are essentially consistent, with only minor differences. However, there is a substantial discrepancy between the method in literature[12] and manual annotation, especially as the enhanced tumor regions are relatively smaller and thus more challenging to segment accurately. The results demonstrate that the method presented in this paper can achieve precise segmentation of brain glioma via 3D-MRI.

**Test the model on additional datasets (e.g., BraTS2020) and consider incorporating multicenter clinical datasets to enhance the study’s real-world applicability.**
Reply: According to the suggestion, the data set used in the subsequent experimental testing has been clarified as follows:

To ensure the applicability of the proposed model, the BratS2020 dataset was used as the test set, and 10 samples of 3D MRI glioma cases were selected. FLAIR modal images have good contrast for observing the boundaries of gliomas and surrounding normal brain tissue. Gliomas often have irregular boundaries and blurry areas between them and surrounding normal brain tissue. FLAIR images can help doctors observe these boundaries more clearly, thereby aiding in accurate segmentation of lesions. Therefore, FLAIR modality MRI images were selected for testing. Images of the 10 samples are presented in Fig 6. To assess the segmentation effectiveness of the proposed method, the highly efficient three-dimensional residual neural network segmentation approach from literature[12] was utilized as a comparative method. This approach segmented FLAIR MRI images of the 3D-MRI brain glioma samples alongside the proposed method, with results compared to manual annotations. Differences in brain glioma segmentation outcomes were analyzed, with experimental results showcased in Figures 7, 8, and 9.

**Fig 6. Original 3D-MRI brain glioma case sample data (a) The 1st 3D-MRI glioma sample (b) The 2nd case of 3D-MRI glioma (c) The 3rd case of 3D-MRI glioma (d) The 4th case of 3D-MRI glioma (e) The 5th case of 3D-MRI glioma (f) The 6th case of 3D-MRI glioma**

**Fig 7. Sample labeling results of 3D-MRI glioma cases (a) The 1st 3D-MRI glioma sample (b) The 2nd case of 3D-MRI glioma (c) The 3rd case of 3D-MRI glioma (d) The 4th case of 3D-MRI glioma (e) The 5th case of 3D-MRI glioma (f) The 6th case of 3D-MRI glioma**

**Fig 8. The segmentation results of 3D-MRI glioma case samples using the method described in this article (a) The 1st 3D-MRI glioma sample (b) The 2nd case of 3D-MRI glioma (c) The 3rd case of 3D-MRI glioma (d) The 4th case of 3D-MRI glioma (e) The 5th case of 3D-MRI glioma (f) The 6th case of 3D-MRI glioma**

**Fig 9. Sample segmentation results of 3D-MRI glioma cases using comparative methods (a) The 1st 3D-MRI glioma sample (b) The 2nd case of 3D-MRI glioma (c) The 3rd case of 3D-MRI glioma (d) The 4th case of 3D-MRI glioma (e) The 5th case of 3D-MRI glioma (f) The 6th case of 3D-MRI glioma**

From the analysis of Fig 7, 8 and 9, it is evident that FLAIR mode MRI segmentation in this study accurately identifies the complete tumor region WT, core tumor region TC, and enhanced tumor region ET in the MRI samples. The enhanced tumor region ET is marked in red, while the core tumor region TC is indicated by a combination of red and blue. The complete tumor region WT encompasses areas labeled in red, blue, and green. In this study's MRI samples, the brain glioma regions for the 1st, 3rd, 7th, and 9th cases were located in the left hemisphere; for the 2nd case, above the right hemisphere; for the 4th, 5th, 8th, and 10th cases, in the right hemisphere; and for the 6th case, in the lower part of the right hemisphere. Notably, the enhanced tumor area in the 10 examples was relatively smaller than other tumor areas, making it susceptible to misclassification. Comparing the FLAIR modal MRI segmentation results of the current method with those in reference[12] and the manually annotated results in Fig 7, the enhanced tumor regions segmented by this study are essentially consistent, with only minor differences. However, there is a substantial discrepancy between the method in literature[12] and manual annotation, especially as the enhanced tumor regions are relatively smaller and thus more challenging to segment accurately. The results demonstrate that the method presented in this paper can achieve precise segmentation of brain glioma via 3D-MRI.

**Deepen Ablation Studies:

Conduct detailed ablation studies to independently quantify the contributions of the ASPP and MFAB modules, and analyze the effects of varying hyperparameters.**
Reply: According to the suggestions made, a large amount of ablation research has been conducted in the article, and the analysis of ASPP and MFAB modules has been provided on page 14 of the article. The 3D U-Net model did not introduce ASPP and MFAB modules, the 3D U-Ne+ASPP model only introduced ASPP modules, and the Improved 3D U-Net model introduced both ASPP and MFAB modules. The specific analysis is as follows:

The efficacy of the brain glioma segmentation model significantly influences its segmentation accuracy. Utilizing the 3D U-Net network as a foundational structure, this paper introduces the ASPP network and a multi-scale fusion attention module, MFAB, to conduct ablation studies. Three brain glioma segmentation network models were developed: the 3D U-Net network model, the 3D U-Net+ASPP network model, and the enhanced 3D U-Net network model. These models were trained using 180 samples from the BraTS2023 dataset([Multimodal Brain Tumor Segmentation Challenge 2023 | CBICA | Perelman School of Medicine at the University of Pennsylvania](https://www.med.upenn.edu/cbica/brats2018/registration.html))[29,30,31], and by comparing the loss changes of each network model in the validation dataset and the test dataset, the performance of each network model was studied. The experimental results are shown in Table 1.

**Table 1. Change analysis of loss function before and after improvement of glioma segmentation model**

| Data set | Training frequency | Losses | | |
| --- | --- | --- | --- | --- |
|  |  | 3D U-Net model | 3D U-Ne+ASPP model | Improved 3D U-Net Model |
| Validate the dataset | 0 | 0.77 | 0.58 | 0.50 |
|  | 80 | 0.58 | 0.39 | 0.18 |
|  | 160 | 0.50 | 0.35 | 0.10 |
|  | 240 | 0.54 | 0.40 | 0.10 |
|  | 320 | 0.59 | 0.43 | 0.10 |
| Test dataset | 0 | 0.78 | 0.65 | 0.52 |
|  | 80 | 0.59 | 0.41 | 0.19 |
|  | 160 | 0.51 | 0.41 | 0.11 |
|  | 240 | 0.50 | 0.36 | 0.11 |
|  | 320 | 0.50 | 0.36 | 0.11 |

According to Table 1, there is a significant difference in the performance of the 3D U-Net network model and the 3D U-Net+ASPP network model between the validation dataset and the test dataset, indicating the presence of overfitting. Among them, in the test dataset, the 3D U-Net network model and the 3D U-Net+ASPP network model showed a decreasing trend with increasing training times. Compared to the losses of the three models, the improved 3D U-Net network model has lower losses than the 3D U-Net network model and the 3D U-Net+ASPP network model. In the test dataset, as the number of iterations increases, the loss of each glioma segmentation network model shows a decreasing trend and ultimately remains stable. The improved 3D U-Net network model showed a rapid decrease in loss during the first 80 iterations, with a significant decrease. After increasing the number of iterations, the model loss exhibited a lower degree of change. When the number of iterations reached 160, the model loss began to stabilize, with a final loss of 0.11, which was not significantly different from the representation in the validation dataset; During the iteration process, the loss of the 3D U-Net network model significantly decreased in the first 80 iterations. Before reaching 260 iterations, the loss value showed a slow downward trend, and the model slowly reached a convergence state after 260 iterations. Its loss was much higher than that of the improved glioma segmentation model, with a loss of 0.50, but there was a significant gap compared to its performance in the validation dataset. The loss of the 3D U-Net+ASPP network model is lower than that of the original 3D U-Net network model due to the introduction of ASPP structure. After 240 iterations, it stabilizes at 0.36, which is significantly different from the performance page in the validation dataset. The experimental results show that, based on the 3D U-Net network model, the introduction of ASPP structure and multi-scale fusion attention module MFAB can effectively learn the features of glioma MRI images from training samples, eliminate the influence of irrelevant information on segmentation results, and introduce additional datasets during training to improve the generalization ability and robustness of the model, thereby enhancing the glioma segmentation effect. This indicates that the improved 3D U-Net network model has outstanding performance.

The impact of different hyperparameters is also analyzed in the article, as described on pages 17-18, as follows:

To assess the impact of different loss functions on glioma segmentation accuracy, three experimental setups were devised using the enhanced 3D U-Net glioma segmentation framework. Scheme 1 employed the Dice loss function; Scheme 2 utilized the Focal loss function; and Scheme 3, the method of this study, combined both the Dice and Focal loss functions. These approaches were applied to segment Whole Tumor (WT), including necrotic and non-enhanced tumors, edema areas, and enhanced tumor areas; Tumor Core (TC), comprising necrotic and non-enhanced tumors, and enhanced tumor areas; and Enhanced Tumor (ET). The changes in DSC, Recall, and Precision metrics were analyzed across these categories. The segmentation effectiveness of this method in different tumor regions and the corresponding results are displayed in Table 5.

**Table 5. Analysis of the influence of the selection of loss function on the segmentation accuracy of glioma**

| Evaluating indicator | Tumor area | Segmentation scheme | | |
| --- | --- | --- | --- | --- |
|  |  | Scheme 1 | Scheme 2 | proposed method |
| DSC | WT | 0.8521 | 0.8603 | 0.9168 |
|  | TC | 0.8043 | 0.8184 | 0.8954 |
|  | ET | 0.7826 | 0.7945 | 0.8674 |
| Recall | WT | 0.8955 | 0.9012 | 0.9426 |
|  | TC | 0.8285 | 0.8499 | 0.9014 |
|  | ET | 0.8011 | 0.8163 | 0.9045 |
| Precision | WT | 0.8915 | 0.8954 | 0.9375 |
|  | TC | 0.8966 | 0.9035 | 0.9369 |
|  | ET | 0.7988 | 0.8345 | 0.9011 |

The analysis of Table 5 indicates that the choice of loss function significantly influences glioma segmentation. Utilizing the enhanced 3D U-Net glioma segmentation structure, this study adopts a combined Dice and Focal loss function approach for segmenting glioma in three distinct regions: WT, TC, and ET. The evaluation metrics - DSC, Recall, and Precision - for this method surpass those of the other two schemes. Specifically, in the WT segmentation of the complete tumor region, the DSC, Recall, and Precision values are 0.9168, 0.9426, and 0.9375, respectively. These are 6.47%, 5.65%, 4.71%, and 4.14%; 4.6%, and 4.21% higher than the corresponding values of the other two schemes. For the TC segmentation of the core tumor area, the values are 0.8954, 0.9014, and 0.9369, showing increases of 9.11%, 7.7%, 7.29%, 5.15%, 4.03%, and 3.34% compared to the other schemes. In the ET segmentation of the enhanced tumor region, the values are 0.8674, 0.9045, and 0.9011, outperforming the other schemes by 8.48%, 7.29%, 10.34%, 8.82%, 10.23%, and 6.66%. This method, integrating Dice and Focal loss functions, not only addresses class imbalance in brain glioma segmentation but also enhances learning of hard-to-classify samples, improving recognition of voxels prone to classification errors and significantly enhancing brain glioma segmentation.

**Strengthen Result Analysis:

Include statistical significance testing and error range reporting to ensure the scientific rigor and reliability of the results.
Provide additional visualizations (e.g., segmentation error maps) and analyze the model’s performance in boundary delineation and noise suppression.**
Reply: Based on the suggestions, we have supplemented the analysis of errors and provided the corresponding segmentation error graph results, as follows:

Based on the above, in order to further verify the performance of the model in boundary delineation and noise suppression, the first and second samples were selected as test samples, and an analysis of segmentation errors was conducted on the red marked enhanced tumor area ET. Under 30dB noise interference, the segmentation error of the proposed model for enhancing tumor area ET in two samples was statistically analyzed, and the expected error was set to 50Pixel The segmentation error results of the proposed model are shown in Figure 11.

**Fig 11. Segmentation Error Results**

According to Figure 11, the proposed method was used to segment the enhanced tumor area ET in the first and second samples, with the highest segmentation error range of 30Pixel to 40Pixel and 18Pixel to 27Pixel, both within the expected range. This indicates that the proposed model has good performance in boundary delineation and noise suppression, with a small error range, ensuring good 3D MRI glioma segmentation results.

**Improve Methodological Novelty:

Consider further optimizing or introducing novel variations to the ASPP and MFAB modules to enhance the originality of the proposed approach.**

Reply: A description of the innovative points of introducing ASPP and MFAB modules is provided in the introduction section, as follows:

this paper introduces an enhanced 3D U-Net network for intelligent segmentation of gliomas in 3D MRI scans. Traditional 3D U-Net networks struggle to capture contextual information of different scales simultaneously when processing brain glioma MRI data, resulting in poor segmentation performance in tumor boundaries and detail areas. This article effectively solves this problem by introducing the Hollow Space Pyramid Pooling Structure (ASPP). ASPP can capture multi-scale contextual information without reducing resolution through multi-scale dilated convolution operations, thereby achieving accurate segmentation of glioma regions. To further enhance the detailed information of gliomas and suppress useless background information, a multi-scale fusion attention module is introduced. This module significantly improves the sensitivity of the network to tumor details through multi-scale feature fusion and attention mechanism, while effectively reducing the interference of background noise, making the segmentation results more accurate and reliable. To tackle the issue of class imbalance and enhance the learning of misclassified voxels, we devise a loss function by combining the Dice loss and Focal loss functions. This approach not only preserves segmentation accuracy but also improves the model's ability to learn from challenging samples, leading to enhanced segmentation performance, precision, and generalization.
