# Supplementary material for: 3D-MRI brain glioma intelligent segmentation based on improved 3D U-net network
Source: PLoS One. 2025 Jun 13;20(6):e0325534. doi: 10.1371/journal.pone.0325534 (PMC12165427; doi:10.1371/journal.pone.0325534)
Supplement: Attachment — Submitted filename: Response_Reviewer_3.docx [file pone.0325534.s004.docx]

Dear Reviewer #3:

Thank you for the reviewer’ comments concerning our manuscript entitled “3D-MRI Brain Glioma Intelligent Segmentation Based on Improved 3D U-Net Network” (ID: 1441728). Those comments are all valuable and very helpful for revising and improving our paper, as well as the important guiding significance to our researches. We have studied comments carefully and have made correction which we hope meet with approval.

Responds to the reviewer’s comments:

Reviewer #3:

We feel great thanks for your professional review work on our article. In the revised draft, your suggestions are marked in red. As you are concerned, there are several problems that need to be addressed. According to your nice suggestions, we have made extensive corrections to our previous draft, the detailed corrections are listed below.

**1. While the authors revised "Validate the dataset" in one paragraph, it still appears unchanged in Table 1 and Table 3. Please ensure consistency throughout the paper.**

**Standardize expressions such as "training set," "validation set," and "test set" across all instances."**

**Data set" should be consistently written as "Dataset."**

**I recommend using an AI proofreading tool, such as DeepSeek or Qwen, to check and correct grammar errors throughout the manuscript.**

Reply: We carefully revised our manuscript. According to your revision suggestions, we made modifications in many places in the manuscript, marked them in red font, and unified "training set," "Dataset," "validation set," and "test set".

**2. The term "Training frequency" appears in Table 1 but lacks explanation. Please clarify its meaning in the table title or description. If it refers to "epoch," consider revising it accordingly.**

**Ensure all terminology in tables and figures is clearly defined within their titles or content.**

**Resolve terminology inconsistencies, such as:"Hollow Space Pyramid Pooling Structure (ASPP)" (page 3) vs. "Atrous Spatial Pyramid Pooling (ASPP)" (page 6).**

**"Evaluating indicator" (Table 5), "Evaluation index" (Section 3.6 title), and "performance metrics" (Section 3.6) should all be standardized as "Evaluation metrics."**

Reply: We made the modification in red font and changed the "training frequency". And we unified the "Atrous Spatial Pyramid Pooling (ASPP)" and "Evaluation metrics" throughout the text.

**3.** **Remove unnecessary background explanations. For instance, Figure 2 (U-Net) should be omitted.**

**Section 3.1 should be condensed into one or two lines.**

**Section 3.6 should be summarized in two lines and merged into Section 4, such as:
"We use Dice coefficient, Hausdorff distance (HD95), recall, and precision for evaluation."**

**"HD distance" is incorrect, as it redundantly means "Hausdorff Distance distance." If using HD95 (the standard metric in the field), explicitly specify it**

Reply: According to your revision suggestions, we made the modifications in red font. We omitted Figure 2 and compressed Section 3.1. We summarize Section3.6 into two lines and merge them into Section 4.We clearly indicated HD95 evaluation metrics.
